# Structure of transcribing RNA polymerase II-nucleosome complex

**DOI:** 10.1038/s41467-018-07870-y

**Published:** 2018-12-21

**Authors:** Lucas Farnung, Seychelle M. Vos, Patrick Cramer

**Affiliations:** Max Planck Institute for Biophysical Chemistry, Department of Molecular Biology, Am Fassberg 11, 37077 Göttingen, Germany

## Abstract

Transcription of eukaryotic protein-coding genes requires passage of RNA polymerase II (Pol II) through nucleosomes, but it is unclear how this is achieved. Here we report the cryo-EM structure of transcribing *Saccharomyces cerevisiae* Pol II engaged with a downstream nucleosome core particle at an overall resolution of 4.4 Å. Pol II and the nucleosome are observed in a defined relative orientation that is not predicted. Pol II contacts both sides of the nucleosome dyad using its clamp head and lobe domains. Structural comparisons reveal that the elongation factors TFIIS, DSIF, NELF, SPT6, and PAF1 complex can be accommodated on the Pol II surface in the presence of the oriented nucleosome. Our results provide a starting point for analysing the mechanisms of chromatin transcription.

## Abstract (web summary)

Eukaryotic transcription requires passage of RNA polymerase II (Pol II) through chromatin, which is impaired by nucleosomes. Here the authors report the cryo-EM structure of transcribing Pol II engaged with a downstream nucleosome core particle at an overall resolution of 4.4 Å, providing insights into the mechanism of chromatin transcription.

## Introduction

The fundamental unit of chromatin is the nucleosome core particle (NCP)^1^, which consists of 145–147 base pairs of DNA wrapped around a histone protein octamer^2^. The NCP poses a significant challenge for polymerases that carry out DNA transcription and replication. Various protein factors enable polymerase progression through NCPs and ensure re-establishment of chromatin after polymerase passage^3,4^. Transcription of eukaryotic protein-coding genes by RNA polymerase (Pol) II is impaired by NCPs^5,6^. Pol II requires transcription elongation factors, chromatin remodelling enzymes and histone chaperones to overcome the nucleosomal barrier and to re-establish chromatin^7–11^. The mechanisms underlying these processes, however, remain poorly understood because Pol II complex structures have only been determined on linear DNA, and not on chromatin templates^12^.

To investigate how Pol II transcribes through a nucleosome, here we reconstitute a complex of transcribing Pol II and an NCP located on downstream DNA, and resolve its structure by cryo-EM. The Pol II–NCP complex structure reveals a defined orientation between Pol II and the NCP, and identifies the contact regions. Comparison with known Pol II complex structures^13–15^ suggests that transcription elongation factors can be accommodated in the presence of the nucleosome. A further comparison with the structure of the chromatin remodelling enzyme Chd1 bound to an NCP^16^ provides a model for Chd1 activation to stimulate Pol II progression through the NCP. After our work was completed, similar structures of NCP complexes with the yeast *Komagataella pastoris* Pol II were reported^17^, which together with our results provide a basis for investigating factor-dependent chromatin transcription.

## Results

### Reconstitution of a transcribing Pol II–NCP complex

To investigate how Pol II passes through chromatin, we determined the structure of Pol II transcribing into a NCP. We initially attempted to obtain structures of elongating Pol II located at different positions within the NCP. We placed transcription bubbles at different NCP locations, but this was unsuccessful. We therefore next reconstituted Pol II elongation complexes on pre-assembled NCPs that were extended with extranucleosomal DNA of different lengths. The extended NCP that enabled structure determination comprised 145 base pairs (bp) of DNA with the Widom 601 sequence, 15 bp of extranucleosomal DNA, a 9-nucleotide (nt) 3′-DNA overhang, and a 10 nt RNA transcript annealed to the DNA overhang (Supplementary Fig. 1a). Pol II can bind the terminal DNA–RNA hybrid duplex of the extended NCP, resulting in a Pol II–NCP complex.

To test whether the reconstituted Pol II–NCP complex could elongate RNA, we incubated the complex with nucleoside triphosphates and transcription factor (TF) IIS, which is known to support Pol II elongation on nucleosomal templates^8^. We observed RNA elongation (Supplementary Fig. 1b), demonstrating that the reconstituted complex was functional. RNA elongation paused at several positions and was almost completely blocked after addition of ~ 35 nt (Supplementary Fig. 1b). The elongation block likely corresponds to the known major pause site around superhelical location (SHL) −5 of the NCP^6^.

### Cryo-EM structure determination

To prepare a sample for cryo-electron microscopy (cryo-EM), we incubated reconstituted Pol II–NCP complex with nucleoside triphosphates. When we subjected the resulting complexes to purification by size exclusion chromatography, TFIIS was lost (Supplementary Fig. 1c, d). The purified sample was examined by single particle cryo-EM (Methods). Particle sorting produced a well-defined Pol II–NCP complex that could be refined to an overall resolution of 4.4 Å. Pol II and the NCP were subjected to multi-body refinement, resulting in a Pol II body at a resolution of 4.3 Å and a NCP body at a resolution of 6.9 Å (Supplementary Fig. 2). The reconstruction clearly shows the nucleic acid sugar-phosphate backbones and major and minor grooves, well-defined secondary structure for Pol II, and the histone octamer fold of the NCP (Supplementary Fig. 3; Supplementary Table 1; Supplementary Movie 1).

We next placed crystal structures of a Pol II elongation complex^18^ (PDB code 3HOV) and an NCP containing Widom 601 DNA^19^ (PDB code 3LZ0) into the cryo-EM density. The structures were locally adjusted to fit the density, connecting DNA was modelled, and the stereochemistry was refined (Methods). Pol II was observed in the active, post-translocated state (Supplementary Fig. 3f). We did not observe additional states of Pol II transcribing into the NCP, despite extensive sorting of the particle images. It is likely these states are unstable and did not survive preparation of the cryo-EM grids. These efforts led to a well-defined Pol II–NCP complex structure.

### Structure of Pol II–NCP complex

**Fig. 1:**
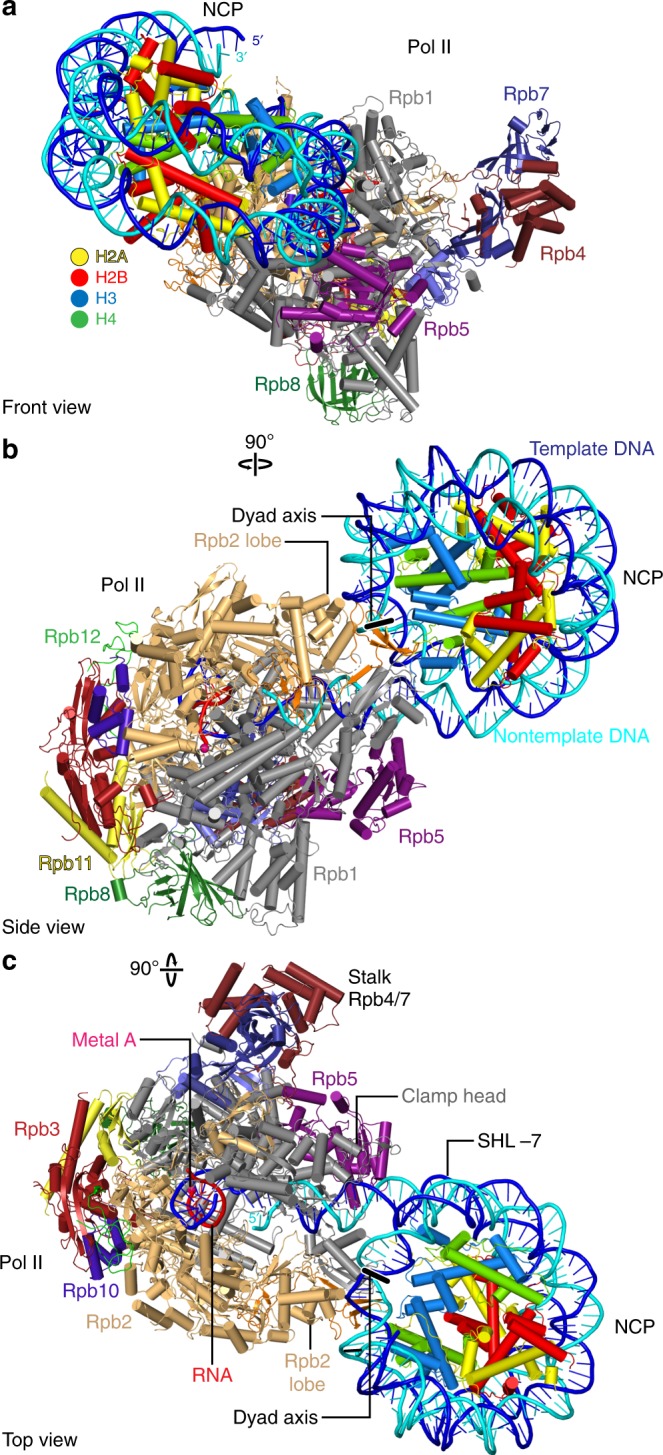
Structure of Pol II–NCP complex. **a**–**c** Cartoon model viewed from the front (**a**), side (**b**), and top (**c**). Pol II subunits Rpb1–Rpb12, template DNA, non-template DNA, RNA, H2A, H2B, H3 and H4 are coloured in silver, sand, ruby, deep purple, slate, cyan, deepblue, forest green, orange, purple, yellow, green, blue, cyan, red, yellow, crimson, light blue and green, respectively. Colour code used throughout. Histone octamer dyad axis is indicated as a black line with white outline

The structure reveals transcribing Pol II engaged with a downstream nucleosome (Fig. 1). The orientation of the NCP with respect to Pol II is defined, and differs from the orientation that could be predicted. To predict the Pol II–NCP orientation, we positioned structures of Pol II and the NCP on the designed nucleic acid scaffold assuming that the connecting DNA adopts the canonical B-form conformation. In this model, the nucleosomal disc is rotated by ~90° compared to the orientation observed in our experimental structure (Supplementary Fig. 4a). Comparison of the model with our structure also reveals that half a turn of nucleosomal DNA has been detached from the histone octamer surface. In particular, DNA base pairs between SHL −7 and SHL −6.5 are lifted from the octamer by up to 5 Å, and this enables feeding of the DNA into the polymerase active site cleft.

**Fig. 2:**
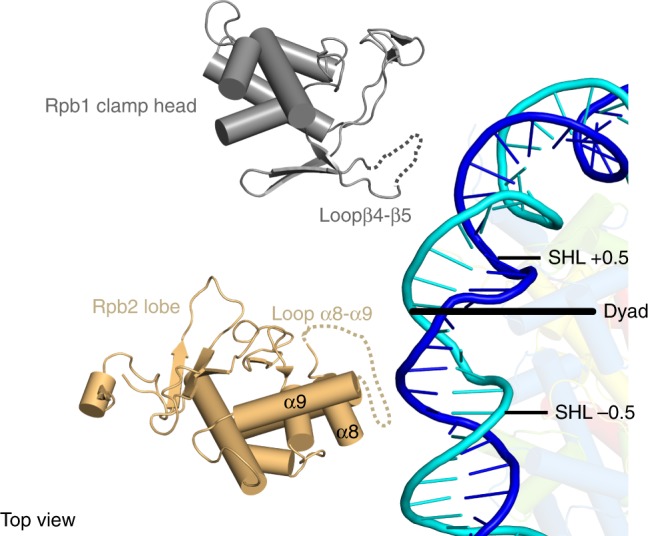
Pol II–NCP contacts. Rpb1 clamp head and Rpb2 lobe domains contact nucleosomal DNA around the NCP dyad. Clamp head and lobe domains are shown. Loops contacting nucleosomal DNA are indicated

These observations indicate that the defined orientation of the NCP results from Pol II–NCP contacts. The structure shows two major contacts between Pol II and the nucleosome (Fig. 2). Two Pol II domains that flank the polymerase cleft contact the NCP around its dyad axis. The clamp head domain of Pol II subunit Rpb1 contacts nucleosomal DNA around SHL +0.5, whereas the lobe domain of Rpb2 forms contacts around SHL −0.5. Pol II–NCP contacts involve the loops β4–β5 (Rpb1 residues 187–194) and α8–α9 (Rpb2 residues 333–345) that protrude from the clamp head and lobe, respectively.

### Model for Pol II pausing near the +1 nucleosome

**Fig. 3:**
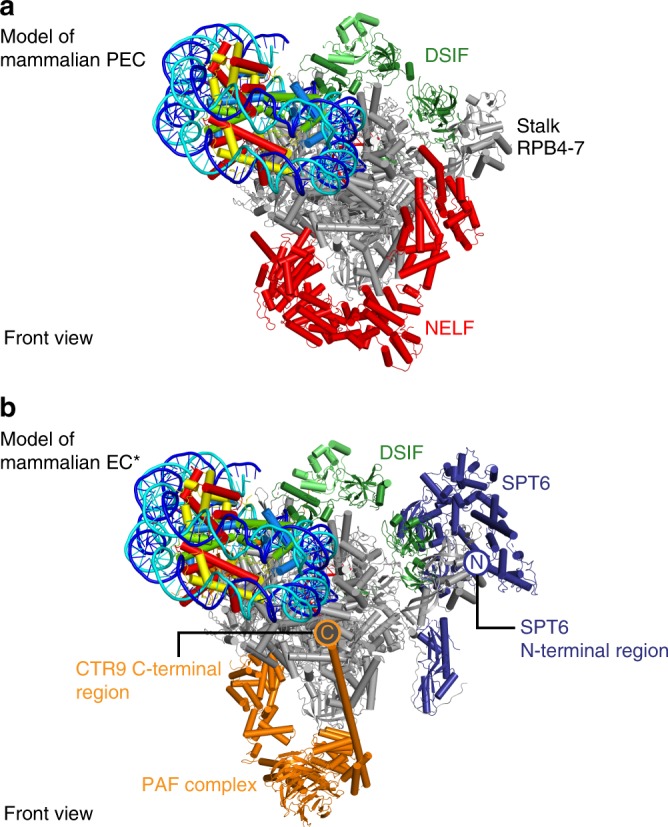
Accommodation of elongation factors. **a** Superposition of Pol II–NCP structure with the mammalian paused elongation complex PEC (PDB code 6GML) provides the location of DSIF and NELF. **b** Superposition of the mammalian activated elongation complex EC* (PDB code 6GMH) provides the location of PAF1 complex (PAF) and SPT6

At the beginning of genes, metazoan Pol II often pauses. The sites of Pol II pausing are generally found upstream of the first nucleosome in the promoter-proximal region (‘+1 nucleosome’). Pausing requires the elongation factors DSIF and NELF, raising the question whether these factors can be accommodated in the presence of the oriented nucleosome. Indeed, superposition of the Pol II–NCP structure onto the structure of the paused elongation complex^15^ reveals that DSIF and NELF can be accommodated on the Pol II surface without clashes with the NCP (Fig. 3a).

The resulting model of paused Pol II located in front of the +1 nucleosome reflects the architecture in vivo. In the fruit fly *Drosophila melanogaster*, the +1 nucleosome adopts a defined location 135–145 bps downstream of the transcription start site^20–22^. This places the upstream edge of the +1 nucleosome at ~60–70 bp downstream of the TSS. Provided that ~15 bp of DNA are accommodated in the Pol II cleft, the active site of paused Pol II would be located 45–55 bp downstream of the TSS, in good agreement with known sites for promoter-proximal pausing^22–24^. We note that the functional relationship between metazoan Pol II pausing and the location of the +1 nucleosome is currently unclear.

### Implications for Pol II passage through nucleosomes

Release of Pol II from pausing and passage of Pol II through nucleosomes requires several protein factors, including TFIIS, the PAF1 complex (PAF) and SPT6^8,25–29^. Superposition with the structure of the activated Pol II complex EC*^14^ shows that PAF and SPT6 can be accommodated in the presence of the oriented NCP (Fig. 3b). In the resulting model, the C-terminus of the PAF subunit CTR9 is in close proximity to SHL –7 of nucleosomal DNA, explaining the known interaction of CTR9 with DNA^30^. The SPT6 N-terminal region is also located near the nucleosome, and is known to interact with the histone chaperone Spn1/IWS1^27,31^, which could bind histones when Pol II progresses and unravels nucleosomal DNA.

Nucleosome passage also requires the ATP-dependent chromatin remodelling enzyme Chd1. To compare our structure with that of the Chd1–NCP complex^16^, we superimposed the NCPs in both structures. The ATPase motor and the double chromodomain of Chd1 may be accommodated on the NCP surface without clashing with Pol II (Supplementary Fig. 4b). However, the DNA-binding region (DBR) of Chd1 must be displaced from its known position on extranucleosomal DNA^16,32^ when transcribing Pol II approaches the NCP. Since displacement of the DBR stimulates Chd1 ATPase activity^33,34^, these observations suggest that approaching Pol II can activate Chd1, which then loosens DNA–histone interactions to aid Pol II progression through the NCP.

Pol II passage through nucleosomes can lead to displacement of one of the two H2A–H2B dimers, converting the histone octamer to a hexamer^35^. The observed Pol II–NCP contacts suggest that the orientation of the NCP is maintained during further Pol II transcription. In this case, the proximal H2A–H2B dimer would encounter the clamp head and lobe domains when Pol II progresses by ~4–5 turns of DNA. The H2A–H2B dimer protrudes from the surface of the nucleosomal disc and would therefore clash with the lobe, possibly leading to its displacement.

## Discussion

Taken together, our structure of a transcribing Pol II–NCP complex reveals a defined orientation between Pol II and the nucleosome, identifies the contact surfaces between Pol II and the nucleosome, and has implications for understanding Pol II passage through the nucleosome. The structure also provides a starting point for analysing the intricate mechanisms of chromatin transcription. In the future, structures of Pol II–NCP complexes with elongation factors such as TFIIS, PAF, SPT6 and CHD1 should be determined. Such studies may be extended to the elongation factor FACT, which is required for transfer of histones from downstream to upstream DNA^7,36^. Ultimately, these studies could show how loss of histones is prevented during Pol II passage, and thus how histone modifications can be retained during transcription.

Others recently reported a similar cryo-EM study of Pol II–NCP complexes using a slightly different experimental strategy^17^. These authors were able to capture Pol II stalled at different positions during transcription into a nucleosome. Comparison of the resulting structures by superposition on Pol II shows that the NCP is in a similar position as observed here. In particular, the NCP position in the complex stalled at SHL −6^17^ is highly similar to that observed in our structure. The published work also reveals similar contacts between Pol II and the NCP. The published study^17^ and our work both raise the puzzling question of how DNA can be further propelled into the Pol II active centre cleft and if the defined Pol II–NCP contacts are maintained as Pol II progresses through the NCP. These studies, however, provide a framework for designing further structural and functional studies to elucidate these intriguing questions.

## Methods

### Protein preparation

*Saccharomyces cerevisiae* Pol II was essentially purified as described^37^. *Xenopus laevis* histones were cloned, expressed and purified as described^16,38,39^. *S. cerevisiae* TFIIS with an N-terminal GST tag was cloned, expressed and purified as described^40^.

### Preparation of extended NCP

DNA fragments for nucleosome reconstitution were generated by polymerase chain reaction (PCR) essentially as described^16,41^. A vector containing the Widom 601 sequence was used as a template for PCR. Large-scale PCR reactions were performed with two PCR primers (CGC TGT TTT CGA ATT TAC CCT TTA TGC GCC GGT ATT GAA CCA CGC TTA TGC CCA GCA TCG TTA ATC GAT GTA TAT ATC TGA CAC GTG CCT, reverse: ATC AGA ATC CCG GTG CCG AG) at a typical scale of 25 mL. The reaction was distributed into 48-well PCR plates (100 μl per well) and PCR was conducted with the following steps: (1) 98 °C for 1 min, (2) 98 °C for 10 s, (3) 72 °C for 45 s, cycle between steps 2 and 3, 35 times, (4) 72 °C for 10 min and (5) pause at 5 °C. PCR products were pooled, phenol chlorofom extracted, ethanol precipitated and resuspended in 1 ml H_2_O. The sample was ethanol precipitated, resuspended in 500 μl H_2_O and digested using TspRI (NEB) to generate the DNA 3′-overhang for RNA binding. Again, the digested DNA was phenol chlorofom extracted, ethanol precipitated and resuspended in 1 ml H_2_O. The DNA was then applied to a ResourceQ 6 ml (GE Healthcare) and eluted with a gradient from 0–100% TE high-salt buffer (10 mM Tris pH 8.0, 1 M NaCl, 1 mM EDTA pH 8.0) over a total volume of 60 mL at a flow rate of 2 mL/min. Peak fractions were analysed on a 1% (v/v) TAE agarose gel and fractions containing the DNA product were pooled, ethanol precipitated, resuspended in 100 µL H_2_O and stored at –20 °C. Preparation of extended NCP was performed as described^16^. Quantification of the reconstituted nucleosome was achieved by measuring absorbance at 280 nm. Molar extinction coefficients were determined for protein and nucleic acid components and were summed to yield a molar extinction coefficient for the reconstituted extended NCP.

### Reconstitution of Pol II–NCP complex

A RNA with the sequence /56-FAM/UCU CAC UGG A was purchased from Integrated DNA Technologies, resuspended in water, and stored at −80 °C. To reconstitute the Pol II–NCP complex, Pol II, extended NCP and RNA were mixed at a molar ratio of 1:0.5:0.66 and incubated for 10 min at 30 °C. Compensation buffer was added after 10 min to a final buffer of 150 mM KCl, 3 mM MgCl_2_, 20 mM Na·HEPES pH 7.5, 4% (v/v) glycerol, 1 mM DTT. For RNA extension, we added TFIIS (1:0.57, relative to Pol II) and NTPs (1 mM final concentration) and incubated for 30 min at 30 °C. The reaction was centrifuged (21,000*g*, 4 °C, 10 min), and applied to a Superose 6 Increase 3.2/300 column equilibrated in gel filtration buffer (100 mM NaCl, 3 mM MgCl_2_, 20 mM Na·HEPES pH 7.5, 4% (v/v) glycerol, 1 mM DTT). Peak fractions were analysed by SDS-PAGE and cross-linked with 0.1% (v/v) glutaraldehyde and incubated for 10 min on ice. The cross-linking reaction was quenched for 10 min using a concentration of 2 mM lysine and 8 mM aspartate. The sample was transferred to a Slide-A-Lyzer MINI Dialysis Unit 20,000 MWCO (Thermo Scientific), and dialysed for 6 h against 600 ml dialysis buffer (100 mM NaCl, 2 mM MgCl_2_, 20 mM Na·HEPES pH 7.4, 20 mM Tris·HCl pH 7.5, 1 mM DTT).

### RNA elongation assays

All concentrations refer to the final concentrations used in 70 µL reactions. Pol II (150 nM) was mixed with linear DNA or extended NCP (75 nM) and RNA primer (100 nM) at 30 °C. Compensation buffer was then added to achieve final assay conditions of 150 mM KCl, 3 mM MgCl_2_, 20 mM Na·HEPES pH 7.5, 4% (v/v) glycerol, 1 mM DTT. TFIIS (90 nM) was added immediately before addition of NTPs. NTPs were added at a final concentration of 1 mM. In all, 10 µL aliquots were taken at 0, 1, 5, 10, 20 and 30 min after NTP addition and were quenched by mixing the sample with 10 µL of Stop Buffer (6.4 M urea, 1× TBE, 50 mM EDTA pH 8.0). Reactions were treated with 4 µg proteinase K for 30 min at 30 °C. The reactions were ethanol precipitated and resuspended in 5 µL of Stop Buffer and applied to a 7 M urea, 13.5% 19:1 Bis-acrylamide, 1× TBE sequencing gel run at 50 W for 35 min in 1× TBE. RNA Products were visualised using the 6-FAM label and a Typhoon 9500 FLA Imager (GE Healthcare Life Sciences).

### Cryo-EM and image processing

The reconstituted and purified Pol II–NCP complex sample was applied to R2/2 gold grids (Quantifoil). The grids were glow-discharged for 100 s before sample application of 2 μl on each side of the grid. The sample was subsequently blotted for 8.5 s and vitrified by plunging into liquid ethane with a Vitrobot Mark IV (FEI Company) operated at 4 °C and 100% humidity. Cryo-EM data were acquired on a Titan Krios transmission electron microscope (FEI/Thermo) operated at 300 keV, equipped with a K2 summit direct detector (Gatan). Automated data acquisition was carried out using FEI EPU software at a nominal magnification of ×130,000. Image stacks of 40 frames were collected in counting mode over 9 s. The dose rate was 5.12 e^−^ per Å^2^ per s for a total dose of 46 e^−^ Å^−2^. A total of 9188 image stacks were collected.

Frames were stacked and subsequently processed. Micrographs were CTF and motion corrected using Warp^42^. Image processing was performed with RELION 3.0-beta 1^43^, unless noted otherwise. Post-processing of refined models was performed with automatic *B*-factor determination in RELION. Particles were picked using the neural network BoxNet2 of Warp, yielding 1,041,545 particle positions. Particles were extracted with a box size of 320^2^ pixel, and normalised. Particles were subdivided into three batches. Using a 30 Å low-pass filtered model from a previously published model (EMDB-3626), we performed iterative rounds of hierarchical 2D and 3D classification with image alignment and activated fast subset option (Supplementary Fig. 2d). Totally, 111,895 particles in batch 1 and 2 that showed Pol II–bound NCP were subjected to masked classification with the mask encompassing the NCP and global 2D classification. Batch 3 also showed Pol II–bound NCP and comprised 44,741 particles that were subjected to a masked 3D classification encompassing the Pol II–NCP complex. Particles from batch 1, 2 and 3 with NCP–bound Pol II were merged, 2D classified, and CTF refined.

The final reconstruction was obtained from a 3D refinement with a mask that encompasses the entire Pol II–NCP complex. The Pol II–NCP reconstruction was obtained from 49,703 particles with an overall resolution of 4.4 Å (gold-standard Fourier shell correlation 0.143 criterion)^44^. The final map was sharpened with a *B*-factor of −182 Å^2^. Similarly to previous yeast Pol II cryo-EM structures, we observed an enrichment of certain Pol II orientations, exemplified in the angular distribution plot^40,45^, but this did not compromise the resolution in any direction (Supplementary Fig. 2c, f). Local resolution estimates were determined using the built-in RELION tool. The Pol II–NCP complex was subsequently multi-body refined using two bodies encompassing Pol II or NCP with an estimated resolution of 4.3 Å for Pol II and 6.9 Å for NCP^46^ (Fourier shell correlation 0.143 criterion).

### Model building

Crystal structures of the *S. cerevisiae* Pol II EC (PDB code 3HOV)^18^, and *X. laevis* nucleosome with Widom 601 sequence^19^ (PDB code 3LZ0), were placed into the density using UCSF Chimera^47^. Extranucleosomal DNA and nucleosomal DNA from SHL −7 to SHL −5 were built using COOT^48^. Secondary structure restraints were applied and the model was real-space refined against the EM maps using PHENIX^49^. Figures were generated using PyMol^50^ and UCSF Chimera and ChimeraX^47^.

### Statistics

No statistical methods were used to predetermine sample size. The experiments were not randomised, and the investigators were not blinded to allocation during experiments and outcome assessment.

## Supplementary information

Supplementary Information

Description of Additional Supplementary Files

Supplementary Movie 1

Reporting Summary

## Data Availability

The cryo-EM density reconstruction and final model were deposited with the Electron Microscopy Data Base (accession code EMD-4429) and with the Protein Data Bank (accession code 6I84). All other relevant data supporting the key findings of this study are available within the Article and its Supplementary Information files or from the corresponding author upon reasonable request. A Reporting Summary for this Article is available as a Supplementary Information file.
