# Supplementary material for: Structure of transcribing RNA polymerase II-nucleosome complex
Source: Nat Commun. 2018 Dec 21;9:5432. doi: 10.1038/s41467-018-07870-y (PMC6303367; doi:10.1038/s41467-018-07870-y)
Supplement: Supplementary file 1 — Supplementary Information [file 41467_2018_7870_MOESM1_ESM.pdf]

Structure of transcribing RNA polymerase II-nucleosome complex

Farnung et al.

Supplementary Data Table 1 | Cryo-EM data collection, refinement and validation statistics

	Pol II-NCP structure (EMDB-4429) (PDB 6I84)
**Data collection and processing**	
Magnification	130,000
Voltage (kV)	300
Electron exposure (e–/Å^2)	46
Defocus range (μm)	0.25-4
Pixel size (Å)	1.05
Symmetry imposed	C1
Initial particle images (no.)	1,041,545
Final particle images (no.)	49,703
Map resolution (Å)	4.4
FSC threshold	0.143
Map resolution range (Å)	4-12
**Refinement**	
Initial model used (PDB code)	3HOV, 3LZ0
Model resolution (Å)	4.5
FSC threshold	0.5
Map sharpening *B* factor (Å^2)	-181.9
Model composition	
Non-hydrogen atoms	43,988
Protein residues	4,665
Nucleotides	337
Ligands	9
B factors (Å^2)	
Protein	98.07
Ligand	107.51
R.m.s. deviations	
Bond lengths (Å)	0.008
Bond angles (°)	1.1
Validation	
MolProbity score	2.16
Clashscore	11.17
Poor rotamers (%)	0.8
Ramachandran plot	
Favored (%)	88.16
Allowed (%)	11.77
Disallowed (%)	0.07

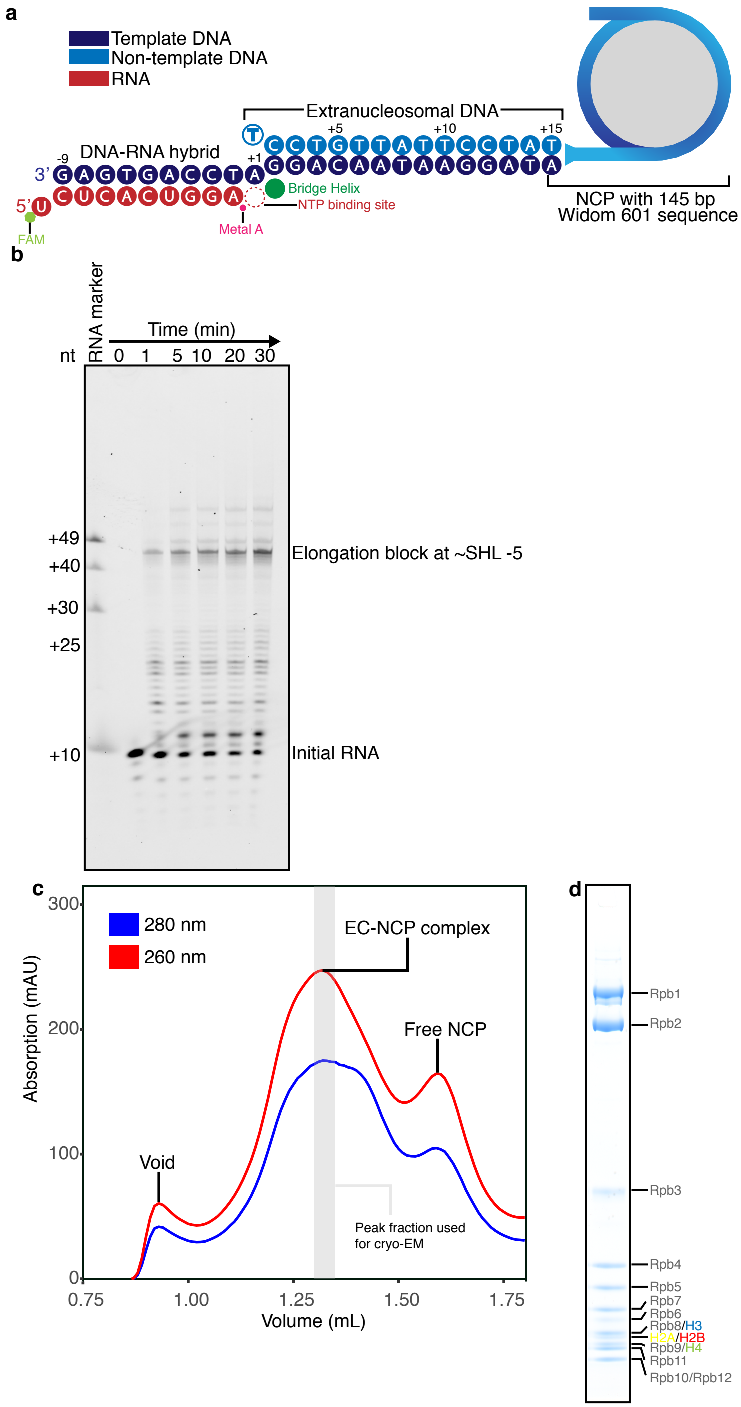

Supplementary Figure 1 | Formation of Pol II-NCP complex.
a, Schematic of nucleic acid scaffold used for RNA elongation assays and complex reconstitution for cryo-EM analysis. **b,** RNA elongation assays performed with Pol II (150 nM), NCP or linear DNA (75 nM), TFIIS (90 nM) with a FAM labelled 10mer RNA primer. Reactions were quenched at various time points. **c,** Formation of the Pol II-NCP complex on a Superose 6 Increase 3.2/30 size exclusion chromatography column. Blue and red curve shows absorption at 280 nm and 260 nm milli absorption units, respectively. **d,** SDS–PAGE of peak fraction from gel filtration (c) used for cryo-EM grid preparation containing Pol II and histones. The identity of the bands was confirmed by mass spectrometry. All experiments were performed at least three times.

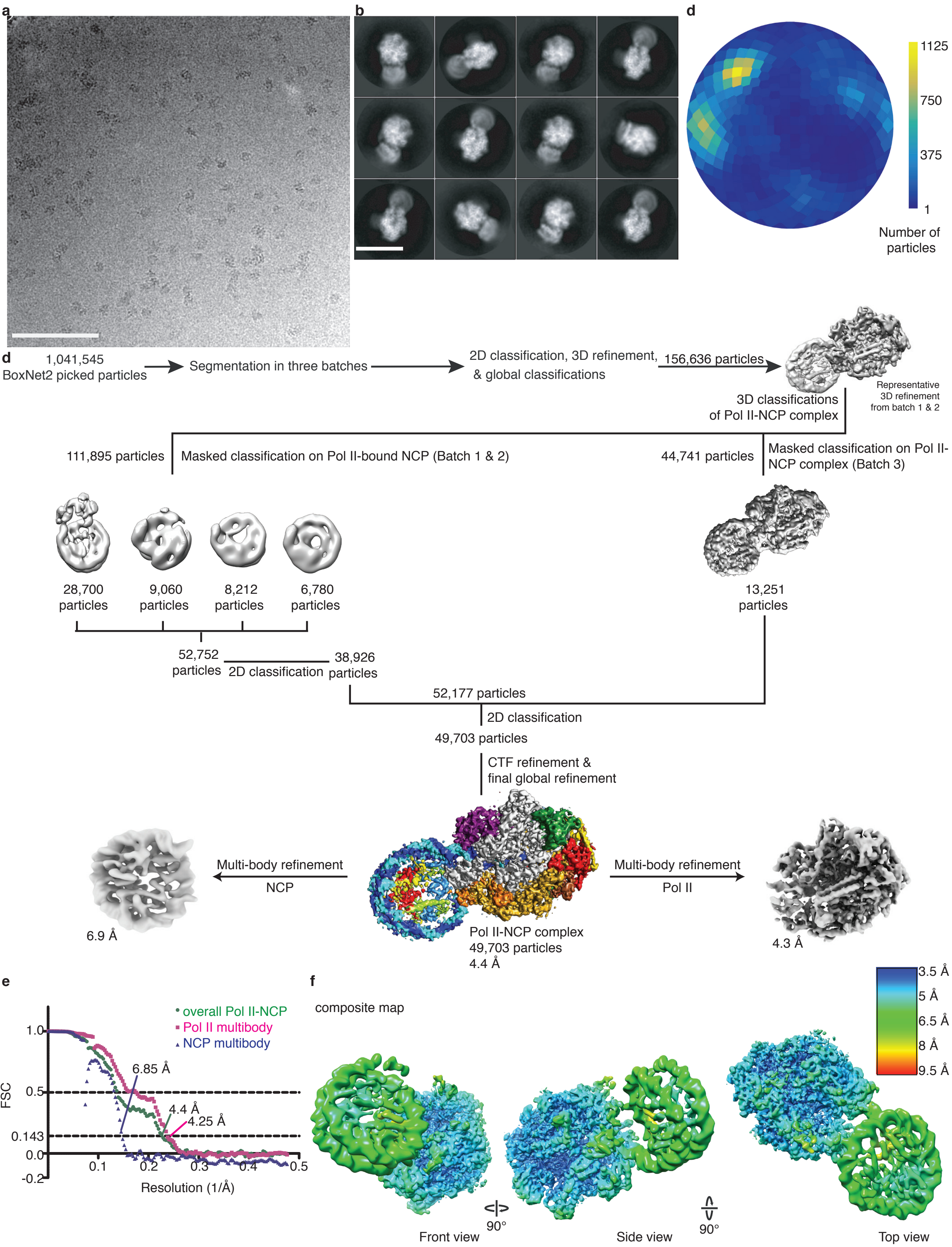

Supplementary Figure 2 I Cryo-EM structure determination
a, Representative micrograph, low pass filtered to enhance contrast. Scale bar is indicated with a length of 100 nm. **b,** 2D class averages show Pol II in close proximity to a nucleosome. Scale bar is indicated with a length of 200 Å. **c,** Angular distribution. Shading from blue to yellow indicates the number of particles at a given orientation. **d,** Sorting and classification tree used to reconstruct the Pol II-NCP complex at 4.4 Å resolution. **e,** Estimation of the resolution. The dark green line indicates the Fourier shell correlation between the half maps of the reconstruction. Resolution is given for the FSC 0.143. **f,** Local resolution estimation. Shading from red to blue indicates the local resolution according to the colour gradient. The multi-body refinement densities are shown. Absolute values are given.

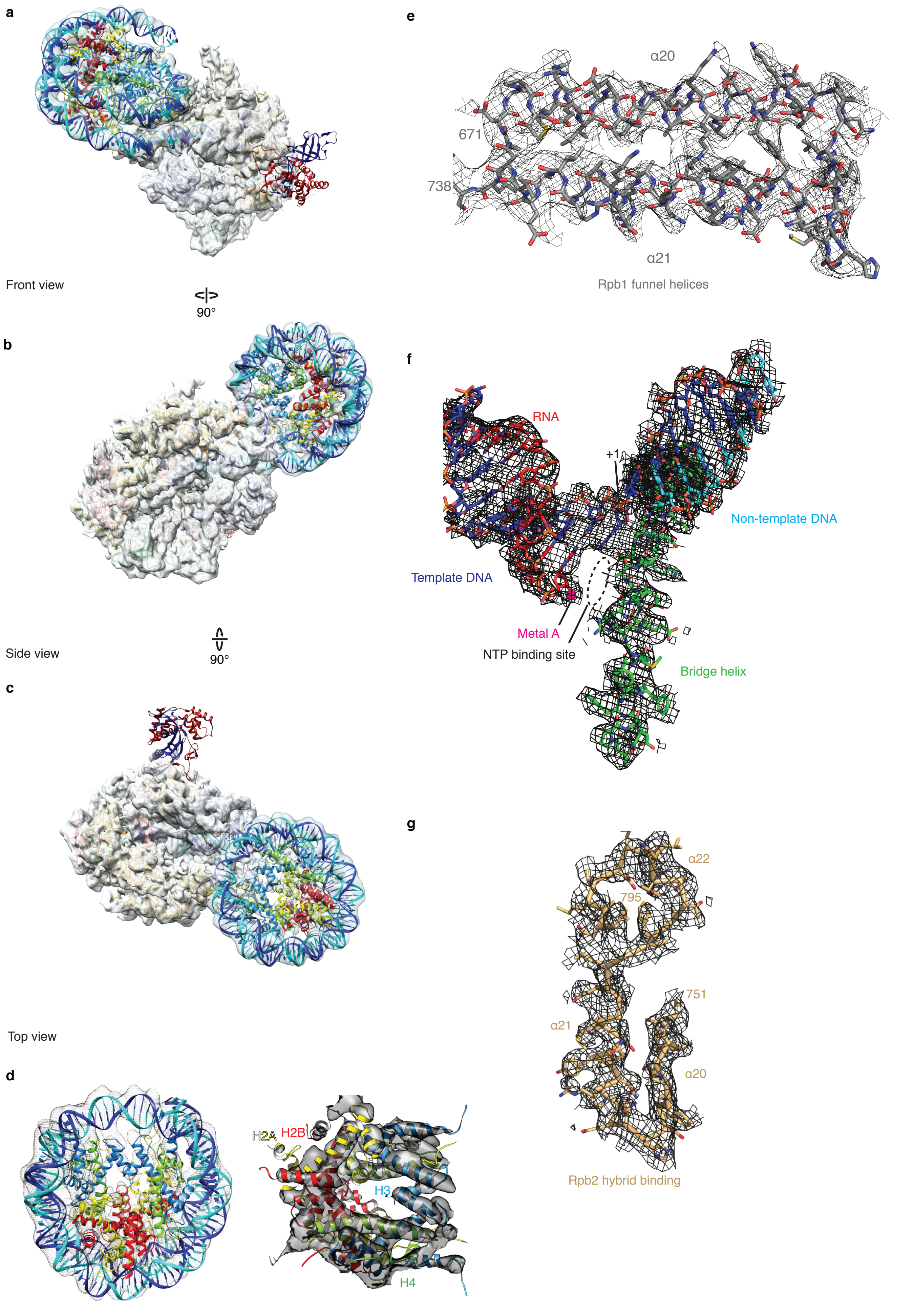

Supplementary Figure 3 | Cryo-EM densities.
a-c, Non-sharpened maps after multibody refinement in **(a)** front, **(b)** side, and **(c)** top views. Pol II density is shown in silver, NCP density in light blue. Rpb4-7 is visible at lower contours. d, NCP density from multi-body refinement resolves the histone octamer fold and nucleosomal DNA. **e,** Rpb1 funnel helices on the Pol II surface are well defined. **f,** DNA-RNA hybrid in the Pol II active site adopts a post-translocated conformation with a free site for the nucleoside triphosphate substrate. **g,** Rpb2 hybrid binding region shows Pol II structure is very well resolved.

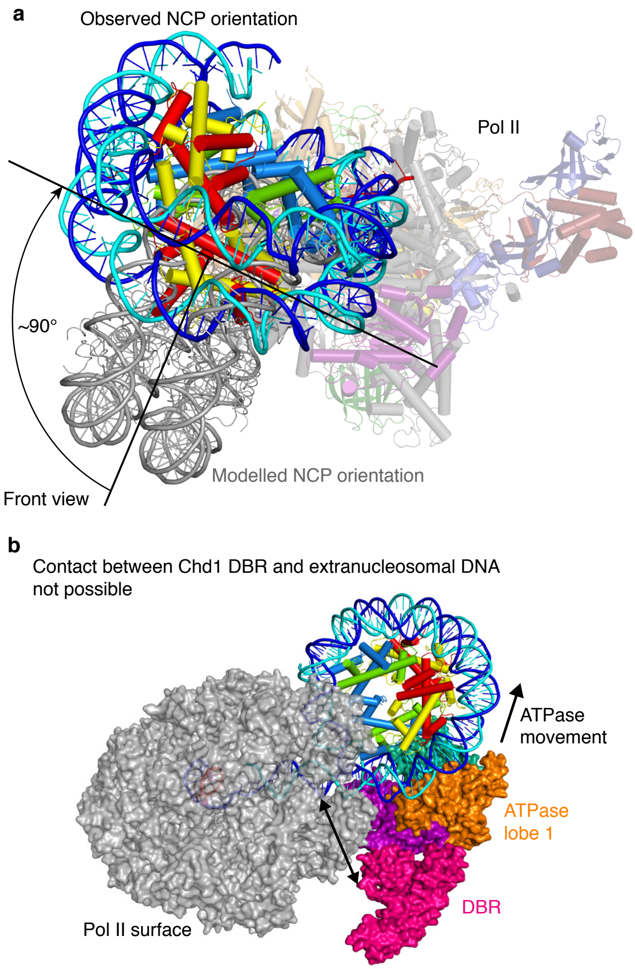

Supplementary Figure 4 I Additional models and structural comparisons.
a, Downstream DNA of a Pol II elongation complex was modelled with a total length of 15 base pairs, followed by a mononucleosome (PDB code 3LZ0, shown in grey) based on our nucleic acid scaffold design. The experimental structure is coloured with the same colouring scheme used throughout the publication. Difference in rotational position of the NCP is indicated. **b,** Superposition with the Chd1-NCP complex (PDB code 5O9G) by aligning the NCPs reveals that the DNA-binding region (DBR) of Chd1 must be displaced from downstream DNA when Pol II approaches the NCP. Chd1 double chromodomain, ATPase lobe 1, ATPase lobe 2, and DNA-binding region are coloured in purple, orange, sea green, and pink, respectively.
