# Supplementary material for: Structure of transcribing RNA polymerase II-nucleosome complex
Source: Nat Commun. 2018 Dec 21;9:5432. doi: 10.1038/s41467-018-07870-y (PMC6303367; doi:10.1038/s41467-018-07870-y)
Supplement: Supplementary file 2 — Description of Additional Supplementary Files [file 41467_2018_7870_MOESM2_ESM.pdf]

Supplementary Movie 1:

Cryo-EM density and structure of the Pol II-NCP complex

Supplementary Table 1:

Cryo-EM data collection, refinement, and validation statistics
